# Protective Roles of Gadd45 and MDM2 in Blueberry Anthocyanins Mediated DNA Repair of Fragmented and Non-Fragmented DNA Damage in UV-Irradiated HepG2 Cells

**DOI:** 10.3390/ijms141121447

**Published:** 2013-10-30

**Authors:** Wei Liu, Xiangyi Lu, Guangyang He, Xiang Gao, Maonian Xu, Jingkai Zhang, Meiling Li, Lifeng Wang, Zhenjing Li, Likui Wang, Cheng Luo

**Affiliations:** 1 Key Laboratory of Food Nutrition and Safety, Ministry of Education, School of Food Engineering and Biotechnology, Tianjin University of Science and Technology, Tianjin 300457, China; E-Mails: liuwei19880216@163.com (W.L.); luxiangyi2013@163.com (X.L.); gaoxiang1084@aliyun.com (X.G.); zhangjingkai521@126.com (J.Z.); limeiling135635@163.com (M.L.); lizhenjing@tust.edu.cn (Z.L.); 2 School of Life Science, Xiamen University, Xiamen 361005, China; E-Mail: he_guangyang@163.com; 3 Department of Food and Environmental Sciences, Division of Food Chemistry, P.O. Box 66 (Agnes Sjöbergin katu 2), Fin-00014 University of Helsinki, Finland; E-Mail: maonian.xu@helsinki.fi; 4 College of Food Science and Engineering, Nanjing University of Finance and Economics, Nanjing 210046, China; E-Mail: wanglifeng_8@163.com; 5 CAS Key Laboratory of Pathogenic Microbiology and Immunology, Institute of Microbiology, Chinese Academy of Sciences, Beijing 100101, China; E-Mail: wonalbert69@gmail.com

**Keywords:** blueberry anthocyanin (BA), cell cycle, DNA damage, UV irradiation, Gadd45, MDM2

## Abstract

Growth Arrest and DNA Damage-inducible 45 (Gadd45) and MDM2 proteins, together with p21 and p53, play important roles in cell cycle checkpoints, DNA repair, and genome integrity maintenance. Gadd45 and MDM2 were activated and transcribed instantly by UV irradiation, whereas blueberry anthocyanins (BA) decreased the gene and protein expression levels in HepG2 cells for up to 24 h, and gradually restored the UV-induced fragmented and non-fragmented DNA damage of the nucleus at a time point of 12 h. Nevertheless, UV-irradiated HepG2 cell arrests occurred mainly in the G1 phase, which indicated G1 as a checkpoint. The proteins, p21 and p53, retain cellular integrity, suppressing the oncogenic transformation by interruption of the G1 phase of the cellular cycle, giving time for repairing the damage to DNA, or apoptosis induction if the damage is too severe to be repaired, while MDM2 and Gadd45 concomitantly ensure the presence of p53 and p21. Thus, we conclude that repair, together with *Gadd45* and *MDM2* genes, were involved in light and dark reaction mechanisms, however, BA could interfere and assist the repair through restoration, although further studies of the complex of the gene cascades triggered and responded to in BA-assisted DNA repair are needed.

## 1. Introduction

When cellular and tissue environments are favorable, cells initiate their division cycle by DNA synthesis and tighten surveillance. Typically, several cell cycle checkpoints are imposed to monitor the accuracy of the cell cycle events, with the option to halt the cell cycle at virtually any transition point if a major malfunction or DNA damage is encountered [1]. Failure to execute these cell cycles accurately signifies that the cell cycle checkpoints may lead to devastating consequences, such as cancer. Checkpoint controls arrest the cell cycle after DNA damage, however, frequently allowing repair to take place before mutations can be perpetuated [2–5]. In multicellular organisms, DNA damage can also induce apoptotic cell death, protecting the organism at the expense of the individual cell [6,7]. Among the cell cycle of three checkpoints, G1 regulation has attracted significant attention in radiobiology.

Harmful environmental factors in carcinogenesis or cancer treatments, including UV irradiation and chemotherapeutic drugs, transiently increase p53 levels to regulate transcription and expression of the downstream gene, p21, subsequently inhibiting cyclin-dependent protein kinase, and block the transition of G1-S by inhibiting the formation of complexes of cyclin E to minimize mutations [8,9]. Growth arrest and DNA damage (Gadd)-45 has been reported to be an important p53-regulated protein for inhibiting DNA synthesis and preventing cells from entering the S phase [10–12]. In addition, Gadd45 also initiates multiple DNA repair processes for an unhelixed broken DNA. MDM2 protein prevents the transcription of the p53 protein by directly binding to the *N*-terminal end of p53, which is a negative feedback regulation of p53 protein in the G1 phase arrest. Therefore, the function of MDM2 protein is to limit the time of the G1 phase arrest and allow repaired cells to re-enter the cell cycle [13]. Gadd45 and MDM2 are decisive for DNA repair. This work is needed to further understand the molecular mechanism of intrinsic and external DNA protection under the intervention of blueberry anthocyanins (BA).

## 2. Results

### 2.1. The Survival Ratios of HepG2 Cells Irradiated at Different UV Dose

**Figure 1:**
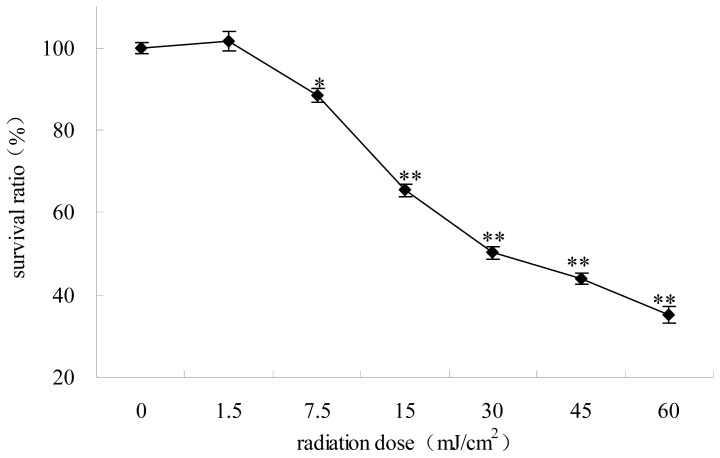
The survival ratios of HepG2 cells irradiated at different UV dose. Each value represents the mean of three experiments. * p < 0.05, ** p < 0.01, compared with control group.

The survival curve by MTT assay after 12 h of cell culture showed nearly 50% viability at the UV radiation dose of 30 mJ/cm^2^, thus this dose was applied in all assays (Figure 1).

### 2.2. The Effects of BA on Morphology of UV-Irradiated HepG2 Cells Observed by SEM

**Figure 2:**
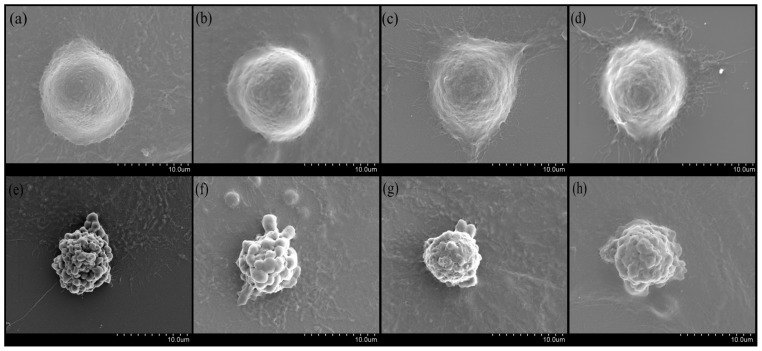
The effects of BA on morphology of UV-irradiated HepG2 cells observed by SEM. Representative photos of cells observed by scanning electron microscopy after pre-treatment for 12 h with BA at different concentrations and then exposed to UV. (**a**) control (without UV radiation); (**b**) 25 μg/mL BA; (**c**) 50 μg/mL BA; (**d**) 75 μg/mL BA; (**e**) UV-radiation without BA; (**f**, **g**, and **h**) were UV+ 25 μg/mL BA, UV+ 50 μg/mL BA, UV+ 75 μg/mL BA, respectively.

In this study, the SEM results demonstrated that the UV-irradiated group appeared wrinkled, curled, and with blisters, whereas BA treatment alleviated UV-extended damage compared with the control cell group, which had cellular integrity and clear surface morphology (Figure 2).

### 2.3. BA Restored UV-Induced DNA Fragmentation in HepG2 Cells

**Figure 3:**
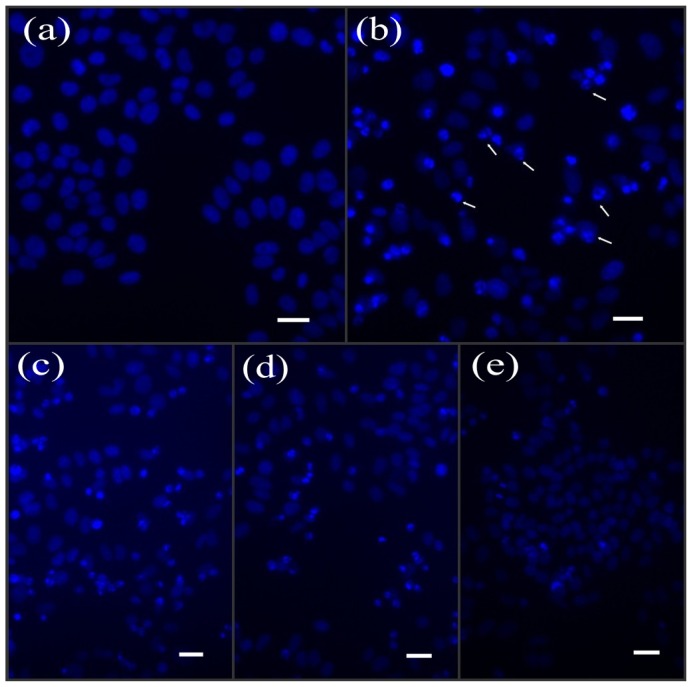
The effects of BA on chromatin condensation in HepG2 cells. Representative images of Hoechst 33258-stained cells observed under fluorescent microscopy after pre-treatment for 12 h with BA at different concentrations and then exposed to UV (scale bar: 50 μM). (**a**) control (without UV radiation); (**b**) UV-radiation without BA; (**c**, **d**, and **e**) were UV+25 μg/mL BA, UV+ 50 μg/mL BA, UV+75 μg/mL BA, respectively.

Upon comparison with the control group, many UV-irradiated cells exhibited brighter nuclear shrinkage that was associated with abnormal DNA chromatin condensation. DNA damage of different degrees (*i.e*., fragmented, non-fragmented and partially fragmented) without BA protection was observed (arrows in Figure 3b). BA at concentrations of 25, 50, and 75 μg/mL reduced the abnormal condensation of DNA in a concentration-dependent manner (Figure 3). The nuclear morphology by using Hoechst 33258 staining was largely consistent with the results of SEM.

### 2.4. The Effects of BA on the Cell Cycle Distribution of UV-Irradiated HepG2 Cells

**Figure 4:**
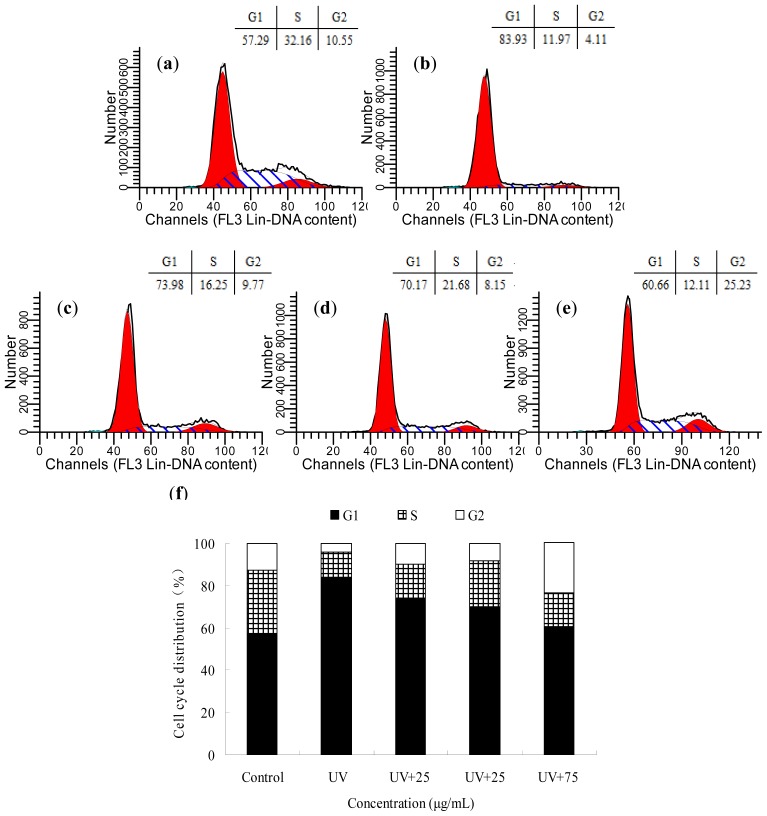
The effects of BA on the cell cycle distribution of UV-irradiated HepG2 cells. After pre-treatment for 12 h with BA, at different concentrations, and then exposed to UV, cell cycle analysis of propidium-iodide stained nuclei was performed by flow cytometry. (**a**) control (without UV radiation); (**b**) UV-radiation without extracts; (**c**, **d**, **e**) were UV+ 25 μg/mL BA, UV+ 50 μg/mL BA, UV+ 75 μg/mL BA, respectively; (**f**) percentage of treated HepG2 cells in varied phase.

The difference in cell cycle distribution under the influence of UV and BA was observed by flow cytometry. Compared with the control group, the fraction of UV-irradiated cells in G1 increased, but the fraction in the S phase decreased (Figure 4b). The BA-treated groups clearly tended to restore cell cycle distribution (Figure 4c,d,e).

### 2.5. Effect of BA on the Membrane Potential of Mitochondria of UV-Irradiated HepG2 Cells

**Figure 5:**
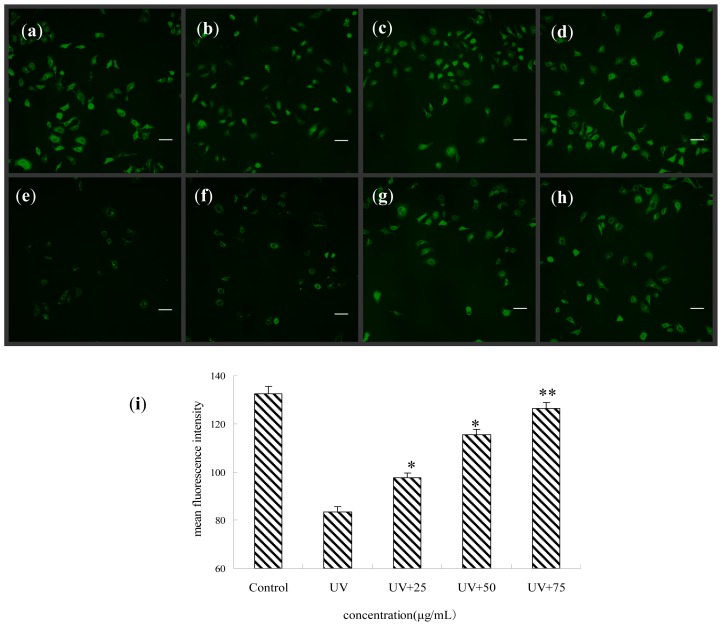
The effects of BA on the membrane potential of mitochondria of UV-irradiated HepG2 cells. Loss of the mitochondrial membrane potential (ΔΨm) was observed at 488 nm (scale bar: 50 μM). (**a**) control (without UV radiation); (**b**) 25 μg/mL BA; (**c**) 50 μg/mL BA; (**d**) 75 μg/mL BA; (**e**) UV-radiation without BA; (**f**, **g**, and **h**) were UV + 25 μg/mL BA, UV+ 50 μg/mL BA, UV + 75 μg/mL BA, respectively; (**i**) the mean fluorescence intensity of 20 cells selected at random was determined. Each value represents the mean of three experiments. * p < 0.05, ** p < 0.01, compared with the UV group.

The mitochondrial membrane potential (ΔΨm) of cells was measured using rhodamine-123. In the control group a striking drop in ΔΨm by mean fluorescence intensity was observed following UV exposure because of mitochondrial membrane depolarization, which is considered to be an initial and irreversible step of apoptosis, whereas the BA-treated group tended to restore the mitochondrial membrane potential (Figure 5).

### 2.6. Protective Effects of BA Extracts on UV-Induced DNA Damage by Comet Assay

**Figure 6:**
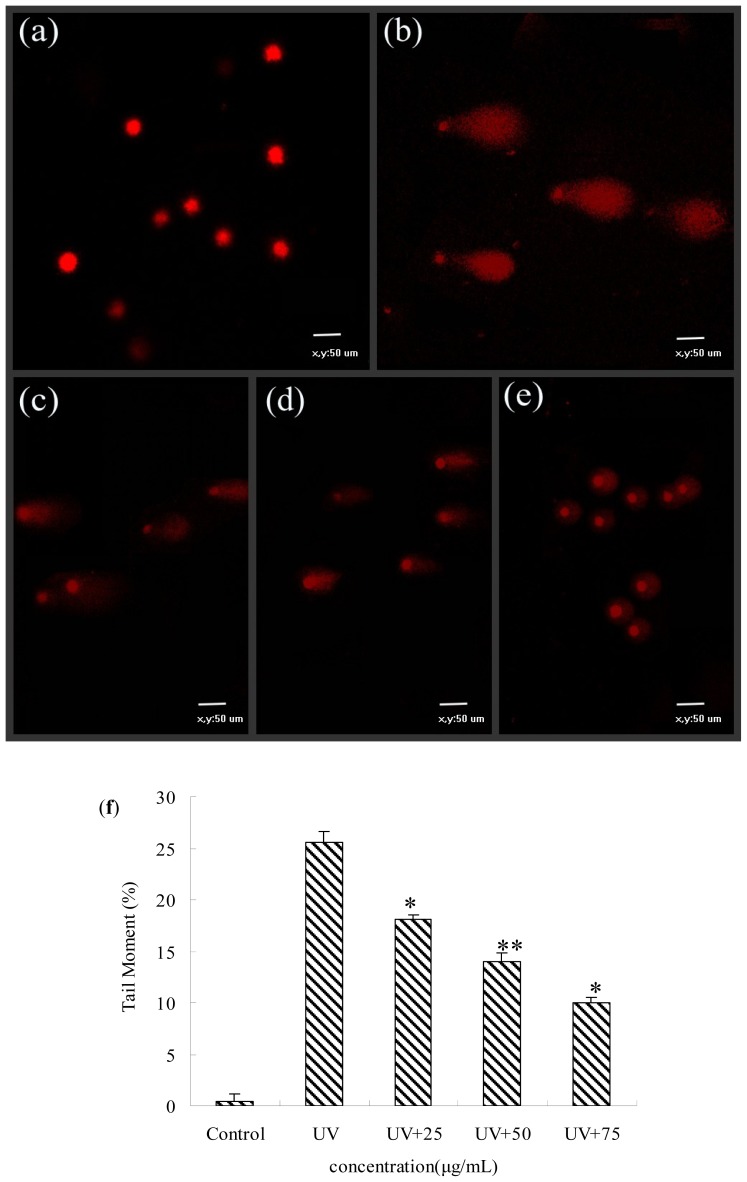
Evaluation of protection by BA extracts against UV-induced DNA damage detected by comet assay. HepG2 cells were pre-treated for 12 h with BA and then exposed to UV. (**a**) control (without UV radiation); (**b**) UV-radiation without extracts; (**c**, **d**, and **e**) were UV+ 25 μg/mL BA, UV+ 50 μg/mL BA, UV+ 75 μg/mL BA; (**f**) quantification of DNA damage was analyzed by CASP software. Each value represents the mean of three experiments. * p < 0.05, ** p < 0.01, compared with the UV group.

Significant protection from DNA damage by BA pre-treatment was also observed by the comet assay using confocal laser scanning microscopy. The fragmented and non-fragmented DNA caused by UV irradiation was demonstrated by comet assay based on the length or the tail moment (Figure 6). The fragmented DNA, especially the small fragments of DNA, can enter agarose gel easily, the more (smaller) fragments, the longer the tail becomes. The DNA fragmented damage by fluorescence of the tail moment was digitally analyzed using CASP software (Figure 6f). These results indicated that pre-treatment with BA significantly decreased the level of DNA damage compared with the control group in a concentration-dependent manner.

### 2.7. Gene Expression of *Gadd45* and *MDM2* Influenced by BA on UV-Irradiated HepG2 Cells

**Figure 7:**
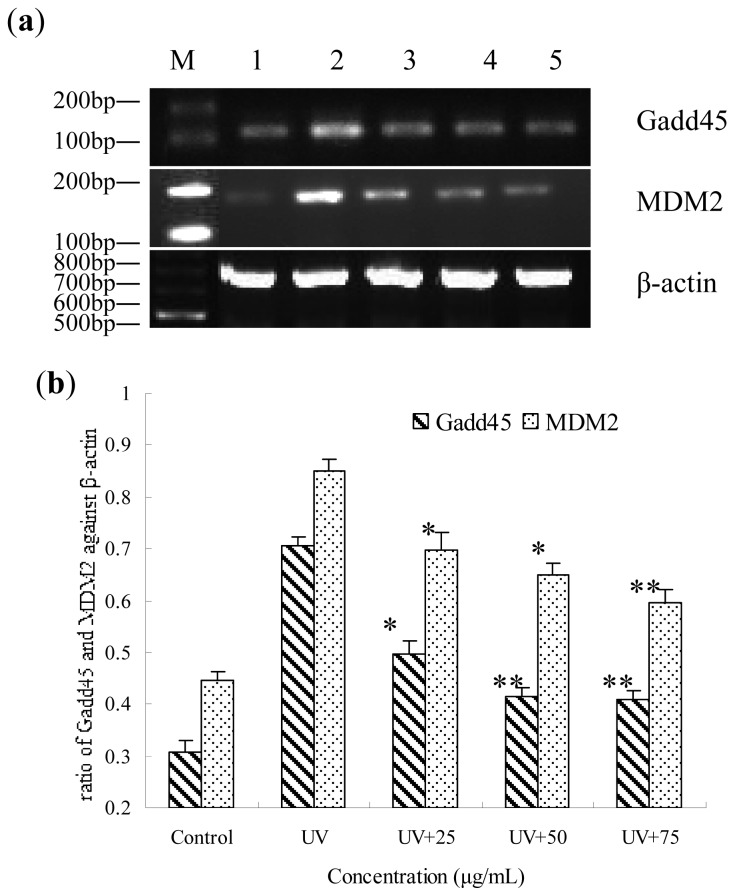
Gene expression of *Gadd45* and *MDM2* by RT-PCR in UV-irradiated HepG2 cells that were pre-treated for 12 h with BA. (**a**) Lane M, marker; lane 1 control (no UV radiation); lane 2, UV radiation; lane 3, UV+ 25 μg/mL; lane 4, UV+ 50 μg/mL; lane 5, UV+ 75 μg/mL; (**b**) The relative expression of *Gadd45* and *MDM2* value after normalizing to β-actin.* p < 0.05, ** p < 0.01, compared with the UV group.

To determine gene regulation in UV-irradiated and BA-treated cells, the expressions of Gadd45 and MDM2 were measured by RT-PCR analysis, and found to be significantly increased in the UV-irradiated HepG2 cells. However, pre-treatment with BA significantly decreased Gadd45 and MDM2 gene expression (Figure 7a,b).

### 2.8. Protein Expression of Gadd45, MDM2, p53 and p21 Influenced by BA on UV-Irradiated HepG2 Cells

**Figure 8:**
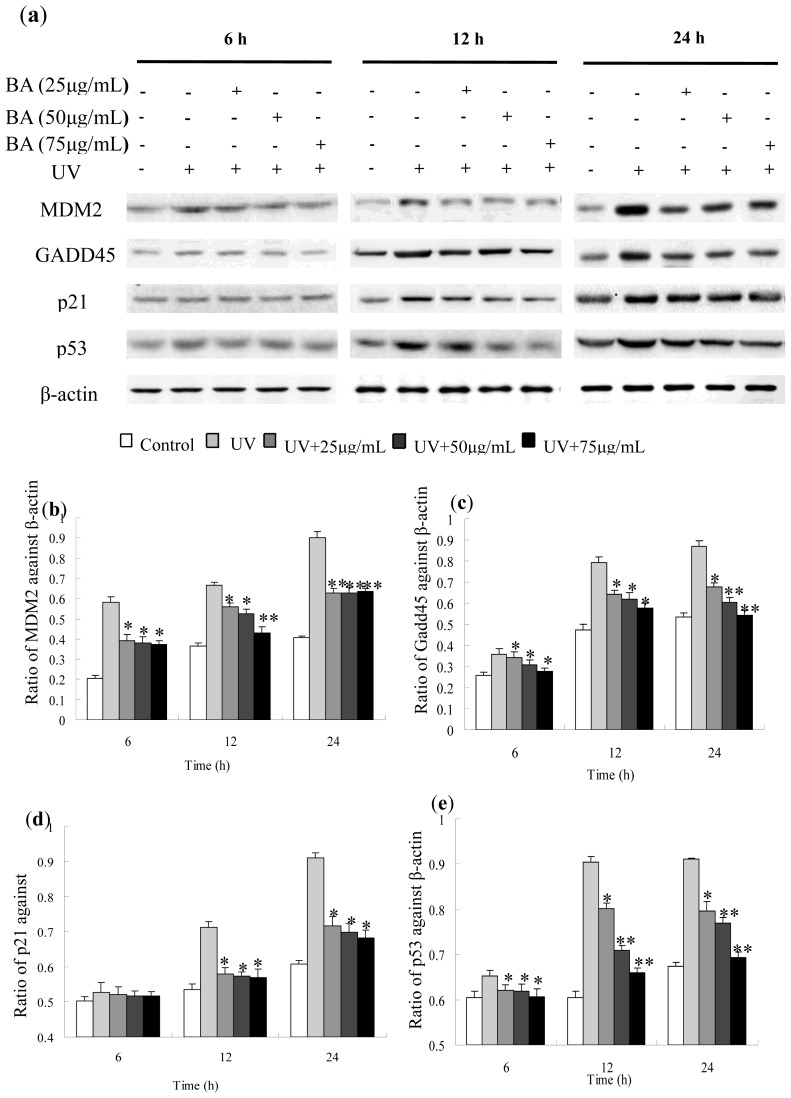
Western blot detection of Gadd45, MDM2, p53 and p21 protein expression in UV-irradiated and BA pre-treated HepG2 cells. (**a**) Western blotting with HepG2 cells that were treated with or without BA, irradiated or non irradiated with a UV dose of 30 mJ/cm^2^ and harvested at the indicated times following treatment; (**b**) The relative expression of Gadd45 value after normalizing to β-actin; (**c**) The relative expression of MDM2 value after normalizing to β-actin; (**d**) The relative expression of p21 value after normalizing to β-actin; (**e**) The relative expression of p53 value after normalizing to β-actin. * *p* < 0.05, ** *p* < 0.01, compared with the UV group.

Expression level of Gadd45, MDM2, p53 and p21 protein measured by Western blotting were consistent with the results of gene expression found by RT-PCR analysis. Moreover, the proteins were persistently present for at least 24 h more after UV irradiation. However, the pre-treatment of BA gradually and significantly decreased the protein expressions of Gadd45, MDM2, p53 and p21 (Figure 8). Equal amounts of total proteins were fractionated by SDS polyacrylamide gels, and Western blots were probed with different Gadd45, MDM2, p53 and p21 antibodies.

## 3. Discussion

Oxidative damage to DNA frequently leads to mutation, and consequently results in different lethal diseases. Base repair in DNA damage is a simple process, but repair of large DNA damage is a complex set of molecular controls in mammalian cells. Cell cycle checkpoints in DNA replication and a complex response of signal transduction of sensor proteins in the network facilitate DNA fidelity [14,15]. In the events responding to double strand DNA breakage and deletion or fragmentation, ATM protein and H2AX are initially triggered and bound to unhelixed DNA between chromatins, where complex of DNA repair machinery should be installed in the correct sequence of events [16]. Signal transducers and pathways influenced during different cell cycles determine cell cycle arrest, DNA repair, or apoptosis [17,18]. The fate of vertebrate somatic cells is decided in the G1 phase of the cell division cycle. The key decisions are whether the cell should proliferate or not, remain, or differentiate. We have reported that blueberry anthocyanins have a protective effect in UV-irradiated cells, which may be related to the antioxidant content of blueberry anthocyanins. We previously found that DNA was significantly damaged after UV irradiation, and the expressions of p53 and p21 proteins were increased, whereas in cells pre-treated with blueberry anthocyanin, p53 and p21 protein expressions were decreased [19], thus, indicating that BA can intervene in the common DNA repair machinery.

UV-irradiated apoptosis and the BA-reversed apoptosis observed in this study occur at early stages and are associated with oxidation and antioxidation [20,21]. The oxidative environment created by the UV dose apparently was not lethal even though the typical apoptotic blebs were observed by SEM. Similarly, DNA fragmentation was observed by the comet assay, but quantities of fragmentation compared with the whole nuclear genome were relatively small. This is also proven by gene and protein expression analysis; Gadd45 and MDM2 were persistently overexpressed beyond 24 h; this made it possible for BA-assisted DNA repair to occur to reverse apoptosis.

It is widely known that p53 is closely associated with DNA damage and repair via regulation of its downstream genes in reaction to DNA damage [22]. When DNA damage is induced by UV light or ionizing radiation, p53 may activate the expression of genes, such as p21 and Gadd45. The expression of Gadd45 allows cells to arrest in the G1/S phase to induce DNA repair and regulate signal pathways of apoptosis and survival [23]. Meanwhile, MDM2 is activated by p53 and then phosphorylated and ubiquitinylated by protein kinase to reduce inhibition of p53 by MDM2. Until the DNA damage is repaired, MDM2 is re-synthesized to inhibit and degrade p53 protein and promote cell cycle to return to normal [24].

This study confirmed that HepG2 cells were arrested in the G1 phase at a dose of 30 mJ/cm2 UV irradiation. Gadd45 and MDM2 proteins were significantly increased 12 h after irradiation and reduced by the intervention of BA. We speculate that the protection of blueberry anthocyanins on DNA damage by UV irradiation is because of its antioxidant activity, ability to scavenge free radicals, and the regulation of relevant DNA repair proteins.

## 4. Experimental Section

### 4.1. Drugs and Antibodies

Dulbecco’s modified Eagle medium (DMEM) and RevertAid First Strand cDNA Synthesis Kit were obtained from Thermo (St. Louis, MO, USA). RNeasy Mini Kit was purchased from Qiagen (Hilden, Germany). Fetal bovine serum (FBS) was obtained from Gibco GRL (Grand Island, NY, USA). Propidium iodide (PI), Hoechst 33258, rhodamine-123, and antibodies of Gadd45, MDM2, p53, p21, and β-actin were purchased from Sigma-Aldrich (St. Louis, MO, USA). Horseradish peroxidase-conjugated secondary antibody and fluorescein-conjugated goat anti-rabbit IgG were purchased from Jackson ImmunoResearch Lab (West Grove, PA, USA).

### 4.2. Isolation and Purification of Blueberry Anthocyanins

Lyophilized blueberries were crushed into powder and defatted with petroleum ether at 80 °C. Extractions of BA were obtained through a Soxhlet extractor at 90 °C for 3 h using 80% (powder/solvent ratio = 1:15 *w*/*v*) ethanol [25]. The extract was filtered through Whatman No. 1 filter paper and evaporated into concentrated solutions in a rotary evaporator at 40 °C. The concentrated solutions were further purified by adsorption of AB-8 macroporous resin, following 60% ethanol for desorption of BA. Finally, the samples were lyophilized to obtain the blueberry anthocyanin extract.

Anthocyanin content was 43.33%, as determined by the pH differential method [26], which is accorded with international regulations that require the anthocyanin content to reach 25% or more to be considered a medical supply material. Structural analysis results show that cyanidin-diglucoside, cyanidin-glucoside, delphinidin-glucoside, and malvidin-galactoside chloride were obtained after the extraction of the blueberry by ethanol from the high performance liquid chromatograph [27].

### 4.3. Cell Culture and UV Irradiation

The extracts of BA were dissolved in dimethyl sulfoxide (DMSO) and diluted in serum-free DMEM to 0, 25, 50, and 75 μg/mL. The final DMSO concentration did not exceed 0.05%, and DMSO was used as a control in each assay. HepG2, a human hepatocarcinoma cell line, was maintained in DMEM supplemented with 10% fetal bovine serum at 37 °C in a 5% (*v*/*v*) CO_2_ incubator.

Irradiation of cells was performed using a standard UV radiation case at a dose of 30 mJ/cm^2^. For synchronization, HepG2 cells were incubated overnight with BA extracts in serum-free DMEM. At the end of the incubation, the medium was removed, and the cells were bathed in serum-free DMEM and then exposed to a fixed UV dose. After irradiation, the cells were incubated for an additional 12 h at 37 °C in an incubator containing 5% CO_2_ prior to use in different assays.

### 4.4. The Survival Ratios of HepG2 Cells Irradiated at Different UV Dose

The MTT assay is a colorimetric assay that measures cell survival [28]. After incubation, the irradiated cells were treated with a 0.5 mg/mL solution of MTT for 3 h at 37 °C. Absorbance was measured at the wavelength of 570 nm. The experiments were performed in triplicate on three separate occasions.

### 4.5. Morphologic Observations

HepG2 cells (5 × 10^6^ cells/mL) were grown on cover slips in 6-well plates. Treated cells were dehydrated in graded concentrations of ethanol [29]. The morphological changes were observed under a scanning electron microscope (SEM) (Hitachi, Tokyo, Japan).

### 4.6. Nuclear Morphology by Hoechst 33258 Staining

HepG2 cells were seeded on coverslips placed in the six-well plates at a density of 1 × 10^4^ cells/cm^2^. The treated cells were stained with Hoechst 33258 staining solution according to the manufacturer’s instructions. The stained cells were examined and photographed under an inverted fluorescence microscope (Seattle, WA, USA) with an excitation wavelength of 350 nm.

### 4.7. Cell Cycle Analysis

HepG2 cells (1 × 10^6^ cells/mL) were seeded in 100 mm dishes. The cells have been synchronized 12 h with serum-free DMEM, then incubated overnight with BA extracts in serum-free DMEM. At the end of the incubation, the medium was removed, and the cells were bathed in serum-free DMEM and then exposed to a fixed UV dose. After irradiation, the cells were incubated for an additional 12 h at 37 °C in an incubator containing 5% CO_2_, then the treated cells were washed in phosphate buffered saline (PBS) and collected by trypsinization, fixed in 70% glacial ethanol, washed in PBS, resuspended in 1 mL of PBS containing 50 U/mL RNase and 50 μg/mL propidium iodide (PI), and then incubated for 40 min in the dark at 4 °C [30]. Cell cycle analysis was performed by flow cytometry (BD, Franklin Lakes, NJ, USA), and the population of cells in each phase was calculated using Modifit LT software. Each experiment was conducted in triplicate.

### 4.8. Analysis of Mitochondrial Potential (ΔΨm)

Changes in mitochondrial membrane potential (ΔΨm) because of mitochondrial dysfunction were detected using fluorescence probe rhodamine-123 [31]. Briefly, HepG2 cells (1 × 10^5^ cells/mL) were incubated in 100 mm dishes. Treated cells were trypsinized and washed with PBS and then incubated at 37 °C for 10 min with rhodamine-123 (5 μg/mL). Finally, the cells were washed twice in PBS and observed by laser confocal scanning microscopy (Nikon, Tokyo, Japan) at an excitation wavelength of 488 nm.

### 4.9. Comet Assay

DNA damage was evaluated through the alkaline single cell gel electrophoresis (comet) assay, performed under alkaline conditions according to previous reports [32] with a slight modification. Cells were plated at a density of 2.2 × 10^6^ cells per dish and left to adhere overnight. Treated cells were then trypsinized, resuspended in PBS and counted. Afterwards, 0.5% normal melting point agarose in PBS (100 μL) prewarmed at 45 °C was dropped onto slides, and they were covered with a glass coverslip. The slides were maintained at 4 °C for 10 min; coverslips were removed, and 10 μL of treated cells were mixed with 75 μL of 0.7% low melting point agarose in PBS at 37 °C and spread onto slides for 10 min at 4 °C. The final layer of 75 μL of 0.7% low melting point agarose was applied in the same way. The slides without coverslips were immersed in frosted lysis buffer with 10% DMSO at 4 °C for 2 h and then placed in electrophoresis buffer (1 mmol/L of EDTA, 300 mmol/L of NaOH, pH > 13) in an electrophoresis tank at 4 °C for 40 min to allow alkaline unwinding. Electrophoresis was performed for 20 min under 25 V and 300 mA. The slides were then transferred to 0.4 mmol Tris-buffer (pH 7.5), washed three times, and gently dried. Comets were stained with propidium iodide (2 μg/mL) and analyzed under a confocal laser scanning microscope (Nikon, Tokyo, Japan). Image analysis and tail moment were performed with CASP 1.2.2 software (Wroclaw, Silesia, Poland); 25 cells were randomly selected per sample.

### 4.10. Gene Expression of *Gadd45* and *MDM2* by RT-PCR

For reverse transcription polymerase chain reaction (RT-PCR) analysis, total RNA was isolated from treated cells using an RNeasy Mini Kit (Qiagen, Hilden, Germany) according to the manufacturer’s instructions. The reverse transcription was performed as follows: 1 μL of Ribolock™ RNase Inhibitor, 1 μL of Oligo(dT)18 Primer, 2 μL of 10 mM dNTP Mix, 4 μL of 5× Reaction Buffer, 2 μL of template RNA (100 ng/μL), and 1 μL of RevertAid™ Reverse Transcriptase were combined. Nuclease-free water was then added to a final volume of 20 μL, mixed, incubated at 42 °C for 1 h and then 70 °C for 5 min for the termination of the reaction. Primers were as follows: Gadd45 with a fragment size of 197 bp: sense 5′-CGAAAGGATGGATAAGGTG-3′ and antisense 5′-GGATCAGGGTGAAGTGGA-3′ based on the template (NM_001924.3); and β-actin with a size of 649 bp: sense 5′-AAATCTGGCACCACACCTT-3′ and antisense 5′-AGCACTGTGTTGGCGTAGAG-3′ based on the template (NG_007992). The PCR reaction protocol consisted of 35 cycles at 94 °C for 30 s, 58 °C for 30 s, and 72 °C for 45 s, followed by a final extension of 10 min at 72 °C. Electrophoresis was performed in 2% agarose gel. Band intensity was quantified with ImageJ Launcher software (National Institutes of Health, Bethesda, MD, USA). The correct PCR fragment was confirmed by a commercial sequencing service company (Biometra, Göttingen, Germany).

### 4.11. Protein Expression of Gadd45, MDM2, p53 and p21 by Western Blotting

Western blotting analysis was performed as previously described with a slight modification [33]. HepG2 cells were treated with BA extracts for 12 h before irradiation. Treated cells were washed with ice-cold PBS and lysed for 10 min in lysis buffer. Equal amounts (60 μg) of protein were separated using 10% SDS-polyacrylamide gels and then transferred to polyvinylidene fluoride (PVDF) membrane. After blotting in 5% nonfat milk in TBST buffer, the membrane was soaked in blocking buffer and incubated overnight with primary antibodies against Gadd45, MDM2, p53 and p21, followed by horseradish peroxidase-conjugated secondary antibodies. Proteins were detected by enhanced chemiluminescence (Amersham, Piscataway, NJ, USA).

### 4.12. Statistics Analysis

All data are presented as the mean ± SEM. Statistical significance of changes in the test responses was assessed using independent samples *t*-test. Significance was assigned at *p* < 0.05. All statistical procedures were performed using SPSS software, version 17.0 (SPSS, Inc., Chicago, IL, USA).

## 5. Conclusions

The molecular weights of BA(s) are relatively small, which may be further metabolized *in vivo*, although this is not the case for the test *in vitro*. There are multiple roles for BA’s intracellular and intercellular functions. However, we may mainly rely on the antioxidative functions at present, which changed the membrane potentials, triggered the proteins (p53/p21 and Gadd45/MDM2) expression differentiation persistently and concomitantly (comparing to controls), reduced radius of comet tail moment and reversed apoptotic blebs, thus we conclude that BA was able to repair the fragmented and nonfragmented DNA damages, and to reverse apoptotic HepG2 cells caused by UV irradiation.
